# Supplementary material for: Effect of Holoptelea integrifolia (Roxb.) Planch. n-Hexane Extract and Its Bioactive Compounds on Wound Healing and Anti-Inflammatory Activity
Source: Molecules. 2022 Dec 4;27(23):8540. doi: 10.3390/molecules27238540 (PMC9738392; doi:10.3390/molecules27238540)
Supplement: Supplementary file 1 [file molecules-27-08540-s001.zip › molecules-2055967-supplementary.pdf]

Supplementary Materials

Effect of *Holoptelea integrifolia* (Roxb.) Planch. *n*-Hexane Extract and Its Bioactive Compounds on Wound Healing and Anti-Inflammatory Activity

Kanokwan Somwong [1,2], Pattawika Lertpatipanpong [2], Wutigri Nimlamool [3], Aussara Panya [1,4], Yingmanee Tragoolpua [1,4], Rujipas Yongsawas [1], Wandee Gritsanapan [5], Hataichanok Pandith [1,4,*] and Seung Joon Baek [2,*]

[1] Department of Biology, Faculty of Science, Chiang Mai University, Chiang Mai 50200, Thailand
[2] Laboratory of Signal Transduction, College of Veterinary Medicine and Research Institute for Veterinary Science, Seoul National University, Seoul 08826, Republic of Korea
[3] Department of Pharmacology, Faculty of Medicine, Chiang Mai University, Chiang Mai 50200, Thailand
[4] Research Center in Bioresources for Agriculture, Industry and Medicine, Chiang Mai 50200, Thailand
[5] Department of Pharmacognosy, Faculty of Pharmacy, Mahidol University, Bangkok 10400, Thailand

*Correspondence: hataichanok064@gmail.com (H.P.); baeksj@snu.ac.kr (S.J.B.)

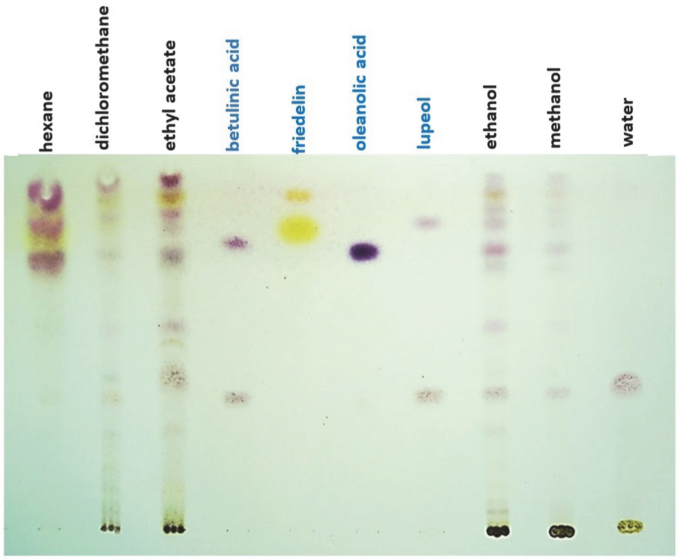

white light

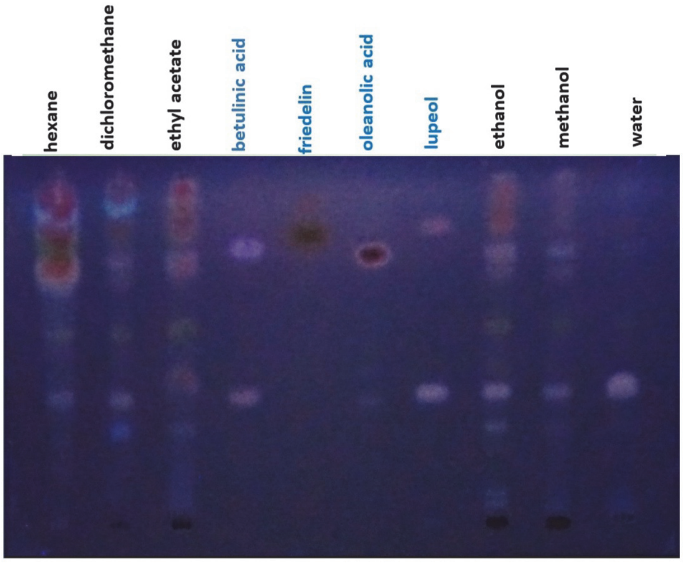

UV light

Supplementary Figure S1. The TLC membrane demonstrated the phytochemical components of *H. integrifolia* extract with various solvents (hexane, dichloromethane, ethyl acetate, ethanol, methanol, and water) were developed along with triterpenoids (betulinic acid, friedelin, oleanolic acid, and lupeol). Hexane demonstrated a promising solvent to show a large amount of phytochemical components.
